# Integrated optical-readout of a high-Q mechanical out-of-plane mode

**DOI:** 10.1038/s41377-022-00966-7

**Published:** 2022-09-28

**Authors:** Jingkun Guo, Simon Gröblacher

**Affiliations:** Kavli Institute of Nanoscience, Department of Quantum Nanoscience, Delft University of Technology, 2628CJ Delft, The Netherlands

**Keywords:** Optical materials and structures, Photonic devices

## Abstract

The rapid development of high-*Q*_M_ macroscopic mechanical resonators has enabled great advances in optomechanics. Further improvements could allow for quantum-limited or quantum-enhanced applications at ambient temperature. Some of the remaining challenges include the integration of high-*Q*_M_ structures on a chip, while simultaneously achieving large coupling strengths through an optical read-out. Here, we present a versatile fabrication method, which allows us to build fully integrated optomechanical structures. We place a photonic crystal cavity directly above a mechanical resonator with high-*Q*_M_ fundamental out-of-plane mode, separated by a small gap. The highly confined optical field has a large overlap with the mechanical mode, enabling strong optomechanical interaction strengths. Furthermore, we implement a novel photonic crystal design, which allows for a very large cavity photon number, a highly important feature for optomechanical experiments and sensor applications. Our versatile approach is not limited to our particular design but allows for integrating an out-of-plane optical read-out into almost any device layout. Additionally, it can be scaled to large arrays and paves the way to realizing quantum experiments and applications with mechanical resonators based on high-*Q*_M_ out-of-plane modes alike.

## Abstract (web summary)

Multilayer structures integrating a photonic crystal cavity and a mechanical resonator with a high-*Q*_M_ out-of-plane mode are fabricated, exhibiting high optomechanical coupling.

## Graphical abstract

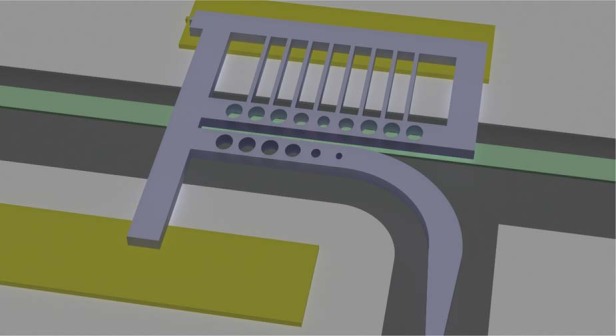

## Introduction

Integrated cavity optomechanical systems have attracted significant attention for their potential use in both classical^1–3^ and quantum^4–6^ applications, and for their ability to study fundamental physics^7,8^. A mechanical resonator with high-quality factor (*Q*_M_) and large coupling to the optical cavity field is desirable, as they are directly related to the ability to maintain coherence and to have efficient read-out and control^9^. Additionally, in order to enable practical applications and advanced quantum experiments, an optomechanical system fully integrated on a chip is required.

In recent years, significant progress has been made in designing and fabricating high-*Q*_M_ integrated mechanical resonators. In particular, out-of-plane mechanical modes with ultra-high-quality factors have been demonstrated^10–14^. Recently, the regime *Q*_M_ *· f*_M_ > *k*_B_*T/h* has been achieved even at room temperature, where *f*_M_ is the resonance frequency of the mechanical mode, *T* is the bath temperature, and *k*_B_ and *h* are the Boltzmann and the Planck constant. Within this regime, the thermal decoherence time is longer than their oscillation periods^9^. This allows to perform quantum-limited sensing^15,16^ or observe macroscopic quantum phenomena^17^ at high temperature, if the mechanical resonator can also be measured in an efficient way^18^. The quantum cooperativity *C*_qu_, which compares the measurement rate to the thermal decoherence rate, and should be around or larger than 1, gives a direct benchmark for reaching the regime of efficient read-out and the potential to perform quantum experiments.

While large mechanical quality factors have been shown in many different systems, coupling these mechanical modes to an integrated optical cavity remains challenging, which limits their potential use in optomechanical applications. To date, there is a disconnect between the largest mechanical quality factors, which are usually out-of-plane modes, and the largest *C*_qu_, which is either achieved in-plane or with bulk-optics setups. In general, an out-of-plane motion has the potential to provide the highest *Q*_M_ *f*_M_ due to the possibility of minimizing the material thickness in the direction of motion^12,19^, which reduces clamping loss, with even the fundamental mode exhibiting high-quality factors^14,20^. Furthermore, out-of-plane modes can achieve a larger surface area perpendicular to the motional direction, which allows for easier coupling to external systems. Both these features make them highly interesting for potential sensing applications^21–24^. Mechanical structures with a high-*Q*_M_ fundamental out-of-plane mode could even help minimize the disturbance from higher order modes, providing a spectrally clean platform for further quantum optomechanical experiments^17,25–27^. Several attempts on making integrated optomechanical devices that couple to the out-of-plane motion have been made^18,28,29^. However, forming a fully integrated optomechanical device with a high-*Q*_M_ mechanical resonator and a large optomechanical coupling remains an outstanding hurdle to practical applications and novel quantum experiments. In contrast, for in-plane mechanical motion, devices based on photonic crystals^26,30,31^ provide a large optomechanical coupling due to the large overlap between the mechanical mode and the confined optical field. This is, however, much more challenging for out-of-plane motion, due to the required small gaps and precision in the photonic crystal structure, combined with multi-layer, 3-dimensional fabrication. The challenging nanofabrication has thus far prevented the realization of such structures.

## Results

In this work, we develop a versatile and flexible new fabrication method enabling the integration of large optomechanical coupling to a high-*Q*_M_ out-of-plane mechanical mode. In particular, we demonstrate devices efficiently coupled to the high-*Q*_M_ fundamental mechanical mode. Our method is based on a pick-and-place technique^32–34^, allowing us to fabricate structures where a photonic crystal (PhC) is placed above a mechanical resonator with a 1.1 MHz fundamental out-of-plane mode and an intrinsic quality factor of around 2 ×10^7^ at room temperature. The resulting devices exhibit a clean mode spectrum around the mode of interest, which in our case is the fundamental mode. This will help to greatly simplify many experiments, as other mechanical modes are at higher frequencies only and are spaced far away. The photonic crystal and the mechanical structure are separated by a controllable small gap of around 100 nm. For spacing of 130 nm, it is possible to achieve an optomechanical coupling rate *g*_0_*/*2*π* ≈ 260 kHz and *Q*_M_ ≈ 1.6 × 10^7^ simultaneously, corresponding to *f*_M_ *· Q*_M_ ≈ 2.9 (*k*_B_*T/h*) at room temperature. We further show that the structure allows to use a large intra-cavity photon number, which leads to a strongly light-enhanced optomechanical coupling *g* and allows to approach unity quantum cooperativity *C*_qu_^9,18^ at high temperature. Our novel technique provides a highly versatile platform for future quantum experiments and applications with high-*Q*_M_ out-of-plane mechanical motion.

**Fig. 1:**
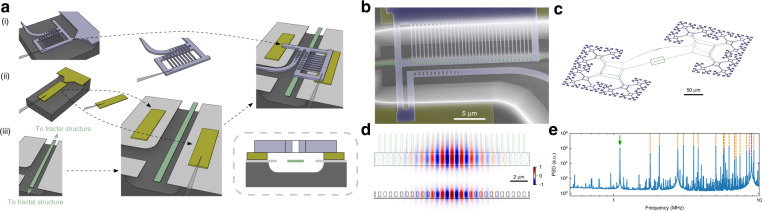
Optomechanical device. **a** Device assembly process. We start with three chips, on which the different structures are fabricated from thin film silicon nitride. They are patterned with (i) a photonic crystal, (ii) spacers, and (iii) center part of a mechanical structure, respectively. A fiber is used to first pick up the spacers and place them on the mechanical chip. Then the photonic crystal is stacked on top of the spacers. The sketch in the box at the bottom-right shows the cross-section of the fully assembled structure. The thickness of the spacers determines the gap distance between the photonic crystal and the mechanical resonator. **b** False-colored SEM image of the assembled device. **c** Simulation of the fundamental out-of-plane mechanical mode of the fractal structure. The mechanical motion is gradually damped from the center to the clamping points. The green dashed box highlights the part of the mechanical structure shown in (**a**) and (**b**). **d** Electric field distribution (top) on the upper surface of the mechanical layer and (bottom) on the center vertical plane. The green dashed line on the upper plot shows the projection of the photonic crystal. The mechanical layer in the simulation has a width of 1 µm, matching the width of the photonic crystal. **e** Measured power spectral density (PSD) of an assembled device, showing how clean the spectrum is around the fundamental mode, with negligible excess noise from other modes. The PSD is measured using a balanced homodyne detection scheme. The green arrow indicates the fundamental high-Q_M_ mechanical mode, the orange dashed lines the higher mechanical modes, while the mechanical mode of the photonic crystal is marked in purple. All other peaks are either electronic noise or mixing between modes in the read-out^31^

The fabrication of our devices is based on a pick-and-place method^33,34^. As shown in Fig. 1a, we first individually fabricate the mechanical and photonic structures, as well as the spacers out of silicon nitride on three separate chips. The spacers are used to provide support to the photonic crystal and to define the gap size between the mechanics and the cavity. The spacers (typical size of 28 µm × 6 µm) and the photonic crystal attach to the original substrate via a weak tether, with a width of about 100 nm. A tapered optical fiber with a sharp tip, fabricated by chemically etching an SMF-28 fiber with hydrofluoric acid^35^, is then placed on the spacers/PhC, which adheres to the fiber through van der Waals and electrostatic forces. By moving the fiber, it is now possible to break the weak tether, while the structure remains attached to the fiber. We first pick up the spacers and position them on the mechanical chip. Then, a photonic crystal structure is transferred and placed above the spacers, as the top layer. When performing the picking and placing, the target chip is on a stage with rotation and three position degrees of freedom. We monitor this process through a camera attached to a microscope. This simple optical imaging is sufficient to achieve good alignment, as can be seen from the optical images taken during the transferring process (Fig. S1) and the scanning electron microscope (SEM) image of the final device (Fig. 1b).

Our mechanical design is inspired by a fractal-like structure^14,20^ (see Fig. 1c), which has been proposed and shown to have an extraordinary high mechanical quality factor of the fundamental mode for low-frequency mechanics. A central string is connected to a block on each side and each unit is then connected to three similar, but smaller, sub (or *child*) units. Our mechanical structure is fabricated from 50 nm thick high-stress silicon nitride, where the tensile stress contributes to the large mechanical quality factor^12,19,36^. In our structure, an additional child unit facilitates preserving the high stress in the parent unit when compared to the binary tree in ref. ^20^. The stress at the center can be maintained or even enhanced without significantly increasing the width of the tethers in the child units. In our structures, the width of the tethers slightly increases from 500 nm at the center to 1.4 µm at the clamping points. In simulations, this results in increased stress of 1.6 GPa at the center tether, up from the 1.3 GPa intrinsic stress. The smaller tether width in our structures further simplifies the silicon undercut process with a fluorine-based dry release method^15,26,37^. In our design, the parent and the child units are connected by a diamond-shaped structure formed by four tethers. Each tether then connects to two other tethers, which is repeated four times to form the full fractal structure (see SI for a detailed sketch). Moving from the center of our structure to the clamping region, the gradient of the mechanical motion displacement can be suppressed gradually at each branching point. The bending is then distributed over the branching regions, forming a softly clamped structure resulting in reduced losses^20^.

The optical read-out cavity is formed by a separate photonic crystal cavity, made from silicon nitride with a width of about 1 µm. The structure and the simulation of the electric field (a quasi-TE mode) are shown in Fig. 1b, d. The photonic crystal cavity strongly confines the light at the center. Extra tethers on the side raise the frequency of its mechanical modes (Fig. S3c), minimizing its impact onto the mechanical noise spectrum. The clamping tethers further improve the thermal conductivity and the rigidity of our photonic crystal, allowing us to use a large cavity photon number without entering the thermal bistability regime^9,38,39^. The evanescent field couples to the mechanical structure, as shown at the bottom of Fig. 1d. By minimizing the distance between the photonic crystal and the mechanical structure, a large optomechanical coupling can be obtained. To further increase the coupling, the part of the mechanical structure below the photonic crystal is widened in order to maximize the overlap with the optical field. In particular, we choose a width of 0.8 and 1.0 µm, similar to the width of the photonic crystal.

A broad-band spectrum, measured after assembling the device, is also shown in Fig. 1e, where the fundamental mechanical mode is marked with a green arrow. Higher order modes are far away from the fundamental mode, where the lowest mechanical peak appears at 1.8 MHz, and the suspended photonic crystal has a fundamental mechanical mode at 9 MHz. Such large relative frequency spacing is extremely challenging to achieve with standard structures with directly integrated phononic crystal cavities^11,12,26^, while it is crucial for many potential experiments^17,25–27^.

**Fig. 2:**
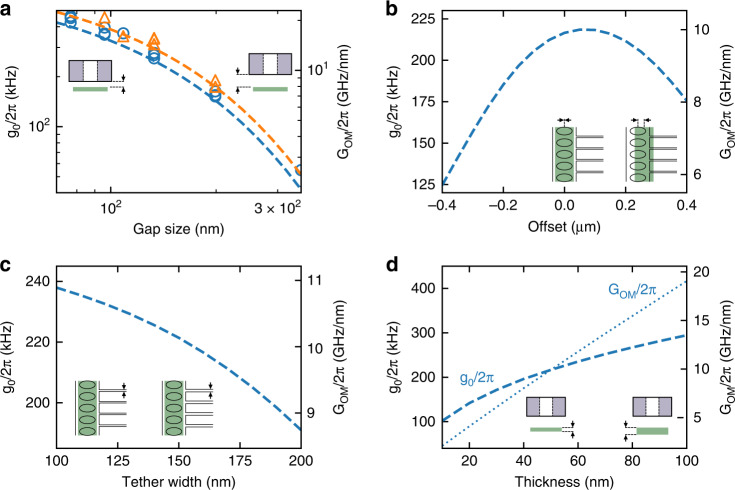
Optomechanical coupling rate. Optomechanical coupling rate *g*_0_ and strength *G*_OM_ for different **a** gap sizes, **b** misalignment or offsets, **c** widths of the clamping tethers, and **d** thickness of the mechanical layer. The dashed lines are simulations, while the circles and triangles in (**a**) are measurement results. The blue and orange color distinguish the different widths of the mechanical part directly under the photonic crystal (0.8 and 1.0 µm, respectively). In (**d**), the change of thickness changes the effective mass. *g*_0_ and *G*_OM_ are plotted separately with a dashed and dotted line, respectively

Achieving a small gap between the mechanical and photonic layers is important to achieve a high optomechanical coupling. This is especially true in our case as the mechanical resonator only couples to the evanescent field from the photonic crystal. We calculate the optomechanical coupling strength *G*_OM_ using the perturbation method^40^ and the corresponding optomechanical coupling rate *g*_0_^9^ for our structure, as shown in Fig. 2. Increasing the gap size from 75 to 350 nm reduces the optomechanical coupling strength *G*_OM_*/*2*π* by one order of magnitude, from 21.6 to 2.2 GHz/nm, for a mechanical resonator width of 1 µm. With our pick-and-place fabrication technique there is, in principle, no real lower limit on the gap size, as it is defined by the spacer. Furthermore, as it is performed in air and no further processing is required afterward, there are no adhesion issues^41^ or risks of collapsing structures. In our setup, we reliably achieve a gap of 75 nm. We compare the measurements on our devices to the simulations of the optomechanical coupling, which shows good agreement. As simulated in Fig. 2b, the misalignment tolerance is also large and our alignment in the lateral direction is sufficiently good to achieve a large optomechanical coupling with this scheme. While a wider PhC structure would allow for an even larger tolerance, it would also lower the mechanical frequency of a low-*Q*_M_ torsional mode (cf. Fig. S3b), which would introduce additional mechanical noise.

In this work, we use an asymmetric design of the photonic crystal, and hence a perfect alignment in the lateral direction (zero offset) does not give the best optomechanical coupling, as the clamping tethers attract the optical field to the side. Thus, a slight offset in our assembly improves the *g*_0_ slightly. The value of the optomechanical coupling is also sensitive to the width of the clamping tethers. As shown in Fig. 2c, when there is no lateral offset, a reduction in the tether width increases the optomechanical coupling. Another factor for the optomechanical coupling is the thickness of the mechanical layer, shown in Fig. 2d. For our structure, *G*_OM_ depends on the electric field difference between the upper and the lower surface of the mechanical structure. This difference is given by the field gradient along the z-direction times the thickness of the SiN layer. As a change in this thickness only has a negligible influence on the electrical field distribution, *G*_OM_ is effectively only proportional to the thickness. The optomechanical coupling rate, *g*_0_, is then proportional to the square root of the thickness due to the change of the effective mass^9,42^ for different thickness. This, however, imposes a trade-off for the thickness of the mechanical resonator since a thinner layer is beneficial for the mechanical quality factor^12,19^.

**Fig. 3:**
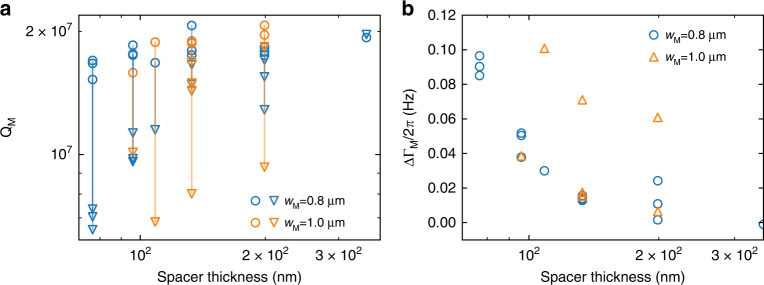
Mechanical quality factor measurements. **a** Measured mechanical quality factor before (circle) and after (triangle) device assembly. **b** Dependence of the change of the mechanical damping rate ∆Γ_M_ on the spacer thickness. A thinner spacer in general leads to a higher increase of dissipation

After assembling the whole structure, we see a slight reduction in the mechanical quality factor compared to the bare resonator, which we independently measure before each integration. Interestingly, this reduction is more pronounced for smaller gaps (Fig. 3). In order to avoid any spurious optomechanical effects in this measurement, we use large laser detunings to determine *Q*_M_. We, therefore, attribute the reduction in quality factor to the coupling of the motion of the mechanical to the optical structure. While the exact mechanism is still under investigation (e.g., electrostatic or van der Waals forces), we can see that by increasing the gap size, the reduction of the quality factor also becomes smaller. An analysis (see Supplementary Information) shows that this reduction depends on the frequency and the quality factor of the mechanical modes of the photonic crystal. As the PhC cavity layer is stress-released, its quality factor is, in general, relatively low (around 500), which is why we design its frequency to be as high as possible (cf. Fig. S3c).

**Fig. 4:**
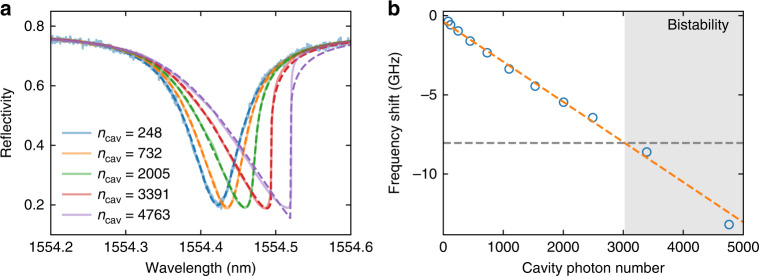
Optical resonance frequency shift for different input power (converted to cavity photon numbers on resonance). **a** Reflection spectrum when sweeping the laser from short wavelength to long wavelength. Dashed lines are fits to extract the frequency shift, and the results are plotted in (**b**). A linear fit is performed to extract the coefficient for the optical resonance tuning, which is used to obtain the bistability bound. The gray dashed line marks the maximal frequency shift above which optical bistability occurs

Being able to use a large intra-cavity photon number *n*_cav_ is important in (quantum) optomechanics, as the single photon coupling rate is enhanced as $g = \sqrt {n_{{{{\mathrm{cav}}}}}} {g}_0^2$. Especially, the quantum cooperativity $C_{{{{\mathrm{qu}}}}} = 4\frac{{n_{{{{\mathrm{cav}}}}}g_0^2}}{{\kappa {{\Gamma }}_{{{\mathrm{M}}}}n_{{{{\mathrm{th}}}}}}}$, where ${{\Gamma }}_{{{\mathrm{M}}}}$ is the mechanical dissipation rate, *κ* is the photon decay rate, and *n*_th_ is the phonon bath number, is a figure of merit for optomechanics in the quantum regime^9^. It compares the photon-phonon interaction rate to the decoherence of the system, and a value comparable to or even higher than unity is required for many experiments^9,43^. Photon absorption and the static optomechanical interaction decreases the optical resonance frequency^42^, which results in optical bistability for sufficiently large photon numbers^38^. This directly limits the achievable quantum cooperativity^9,44–46^. By introducing the additional clamping tethers on the photonic crystal, we also increase the thermal anchoring to the environment and show that it is possible to achieve large *n*_cav_ before entering the bistability regime. We slowly sweep the laser across the optical resonance from short to long wavelengths at various input powers and measure the reflection. The measurements are shown in Fig. 4, for a device with *κ*/2π = 10.1 GHz, gap size 130 nm, and *Q*_M_ = 1.49 × 10^7^. As the input power is increased, the reflection signal becomes more and more asymmetric. We fit the reflection curves and, from the asymmetry, extract the cavity resonance frequency shift. For this device, we find that the bistability occurs for *n*_cav_ ≥ 3000, corresponding to *C*_qu_≈ 0.2 at room temperature, which is right around the regime required for quantum experiments^9,43^ and several orders of magnitude higher than in previous experiments^18,26,44^. While typically, the mechanical quality increases significantly when lowering the temperature^14,36^, already conservatively assuming no change in *Q*_M_, operating at 77 K will lead to *C*_qu_ ≈ 1.

## Discussion

In conclusion, we have demonstrated a simple yet highly versatile technique to integrate a photonic crystal cavity with a mechanical device, realizing large optomechanical coupling between the high-*Q*_M_ out-of-plane fundamental mechanical mode and the optical read-out field. This is achieved by picking spacers and photonic crystals from two separate chips and by placing them onto the chip with the mechanical resonator. The process is robust against misalignment, while the optomechanical coupling strength is sensitive to the distance between the photonic crystal and the mechanical layer, which we can easily set by using spacers with the desired thickness. Interestingly, the gap size influences the mechanical quality factor, which likely stems from a non-optical coupling between the mechanical motion of the two layers. Our new approach paves the way to integrate almost any mechanical design with an on-chip efficient optical read-out scheme. In our demonstration, we use a system with large mechanical quality and a clean mode spectrum, which makes it highly interesting for various applications^17,25–27^. Furthermore, we demonstrate that, with our newly designed photonic crystals with clamping tethers, a large intra-cavity photon number can be achieved, leading to close to unity quantum cooperativity at room temperatures. This will allow for realizing quantum optomechanical experiments and practical quantum sensor applications with no, or only very modest, cryogenic pre-cooling.

## Materials and methods

A detailed description of the methods can be found in the Supplementary Information.

## Supplementary information

Supplementary Information

## Data Availability

Source data for the plots are available on Zenodo at 10.5281/zenodo.7091564.
