# Supplementary material for: Integrated optical-readout of a high-Q mechanical out-of-plane mode
Source: Light Sci Appl. 2022 Sep 28;11:282. doi: 10.1038/s41377-022-00966-7 (PMC9519924; doi:10.1038/s41377-022-00966-7)
Supplement: Supplementary file 1 — Supplementary Information [file 41377_2022_966_MOESM1_ESM.pdf]

Supplemetary Information for:
Integrated optical-readout of a high-Q mechanical out-of-plane mode

Jingkun Guo and Simon Gröblacher[1,*]

[1] *Kavli Institute of Nanoscience, Department of Quantum Nanoscience, Delft University of Technology, 2628CJ Delft, The Netherlands*

Microfabrication

All of our chips are based on a high-stress silicon nitride film deposited on silicon in an LPCVD furnace. First, the chips are spin-coated with resist, after which a standard electron beam lithography process is performed. The pattern generated by the lithography is then transferred into the silicon nitride film by using a CHF_3 plasma etch. Next, the resist is washed away and the chip cleaned in piranha solution, followed by hydrofluoric acid dip to remove any oxidation. Finally, we release our structure by performing a fluorine-based etching process [1–3].

Assembling setup and procedure

The chip is placed on a piezo-controlled motorized 3-axis stage, which itself is mounted on a stage whose rotational and tilted angle can be manually tuned. The chip is viewed from the top by a camera through a 50× objective. A single-ended conical optical fiber taper [4] is placed between the objective and the chip, and it is mounted on a motorized z-stage. The setup is enclosed in a box to avoid disturbance due to the air flow in the lab.

The optical images of the full assembling procedure of a device are shown in Figure S1, with the top rows showing the pick-and-place of the spacer, while the bottom row shows the same transfer process for the photonic crystal. In order to start the assembly, a chip with the spacers, suspended and weakly attached to the substrate, is first placed under the microscope objective (50×) and monitored by a camera. An optical fiber is then used to bend the weak connection between the spacer and the substrate until the connection breaks. The spacer is then positioned above the mechanical chip. Rotational alignment is done beforehand by fitting the edge of the spacer and by comparing it to alignment markers close to the mechanical structure (not shown). The chip is then raised slowly until the spacer touches down. A change in color is visible when this process is completed. These steps are repeated for several spacers. The transfer of the photonic crystal follows the same procedure with the fiber only touching the side of the photonic crystal, avoiding any contamination or damage. Note that for a large photonic crystal structure, not all of it touches down simultaneously. This is noticeable from the color difference for various parts in Figure S1(e). In this case, an additional step is performed to push down the other parts of the photonic crystal in order to stick onto the spacers. A fully assembled structure is shown in Figure S2.

In our process, we first measure the quality factor of the mechanical devices. There is a small variation of the bare quality factor of around 15%. We then perform the assembling procedure described above, with an overall yield of more than 75%. Unsuccessful assembling typically includes large misalignment, sticking between the photonic crystal and the mechanical structure, and a non-flat contact surface between pads and the photonic crystal, which is probably caused by dust. Any unsuccessful assembly can be directly seen with our camera, and these devices are not measured afterwards. Data for all the measured devices during this work are presented in Figure 2 and Figure 3 in the main text.

We would like to note that occasionally the fiber tip used in the assembly process can get damaged and become relatively blunt (see for example Figure S1). This does however not affect the pick-and-place procedure in any way, as long as the fiber tip remains smaller than the structure itself.

Reduction of mechanical quality factor

As mentioned in the main text, we typically see a small reduction of the mechanical quality factor after the full structure is assembled. While the exact cause is still not fully understood, we can attribute it to the coupling between the mechanical structure and the photonic crystal, mediated either by charges on the structures, or by van der Waals forces. As the fundamental mechanical mode of the photonic crystal (shown in Figure S3(c)) has relatively low Q_{M}, this results in an additional loss channel for the mechanical structure.

If we label the displacement of the photonic crystal as δz_{PhC}, and the displacement of the mechanical resonator as δz_{M}, we can write the general coupling between the structures as

$$F^c_{\mathrm{PhC}} = -F^c_{\mathrm{M}} \approx F^c_0 + \alpha_c(\delta z_{\mathrm{PhC}} - \delta z_{\mathrm{M}}). \qquad \text{(S1)}$$

* s.groeblacher@tudelft.nl

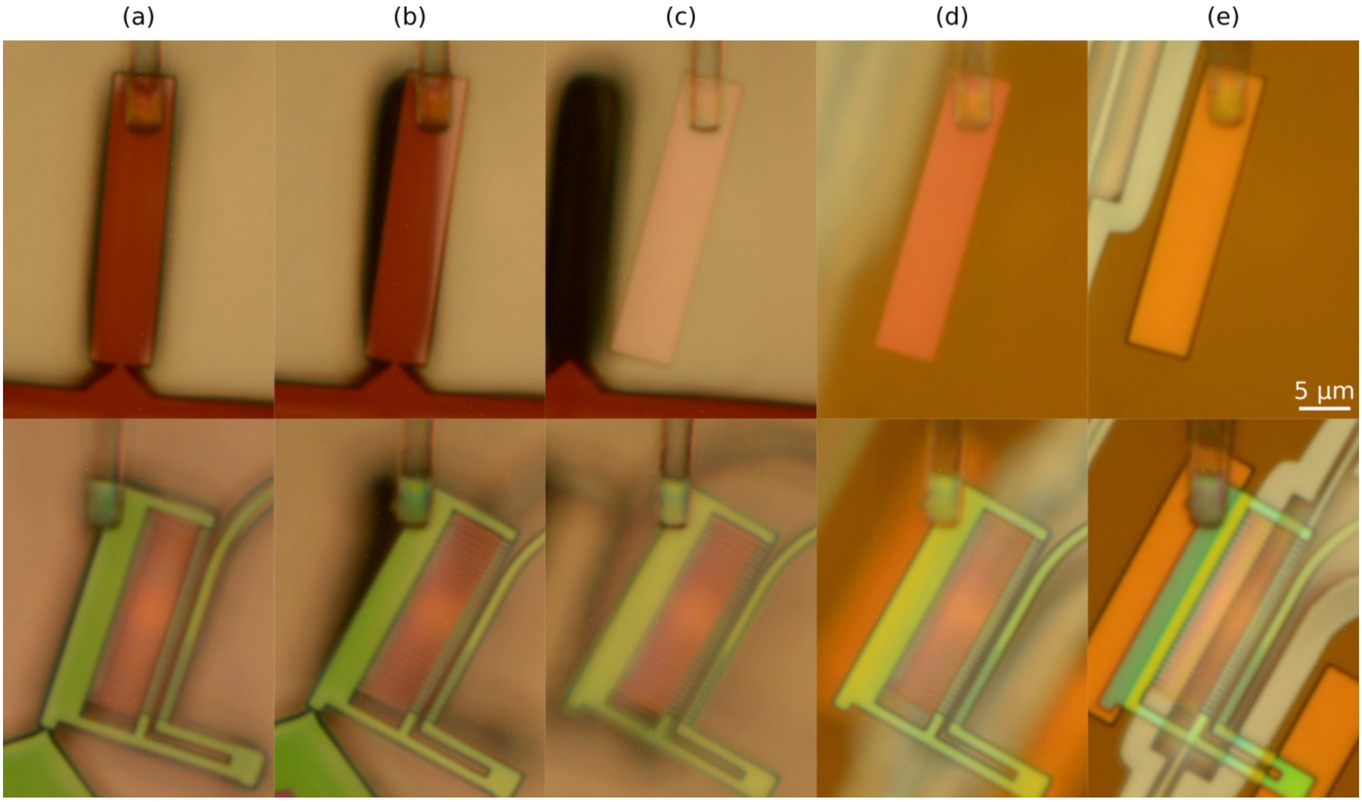

FIG. S1. Device assembling procedure for the spacers (top) and the photonic crystal (bottom).

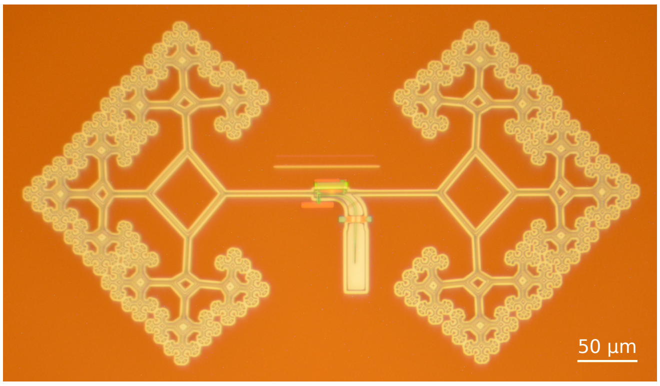

FIG. S2. Optical image of an assembled device.

Here, F^c_{PhC} is the force exerted from the mechanical resonator on the photonic crystal, and F^c_{M} is the force on the mechanical resonator. F^c_0 is the force at equilibrium, and α_c is the linear coupling strength. Higher order terms to the displacement are neglected for small displacement. As we are only interested in the dynamical behavior, we can neglect F^c_0 as it only introduces a constant shift for the equilibrium position.

We further approximate the mechanical resonator and the mechanical motion of the photonic crystal as damped harmonic oscillators and write their equations of motion in the Fourier domain

$$\begin{aligned}\chi^{-1}_{\mathrm{PhC}}(\omega)\delta z_{\mathrm{PhC}}(\omega) &= \alpha_c(\delta z_{\mathrm{PhC}}(\omega) - \delta z_{\mathrm{M}}(\omega)) + F_{\mathrm{PhC}}(\omega),\\ \chi^{-1}_{\mathrm{M}}(\omega)\delta z_{\mathrm{M}}(\omega) &= -\alpha_c(\delta z_{\mathrm{PhC}}(\omega) - \delta z_{\mathrm{M}}(\omega)) + F_{\mathrm{M}}(\omega).\end{aligned} \tag{S2}$$

$$\chi_j^{-1}(\omega) = m_j\left(\Omega_j^2 - \omega^2 - \mathrm{i}\Gamma_j\omega\right) \tag{S3}$$

is the inverse of the susceptibility of the mechanical resonator ($j = \mathrm{M}$) and of the photonic crystal ($j = \mathrm{PhC}$). F_j is the force from other sources, such as the thermal force. In a practical structure, multiple modes present. For the specific geometry of the photonic crystal, the three lowest order modes do not have a large frequency difference and thus our simplified analysis only serve to understand the behavior phenomenologically. We further acknowledge that the dissipation Γ_j is also frequency-dependent for internal damping [5–7]. As we will only be focusing on a small frequency range around Ω_{M} we drop this frequency dependency. For a purely internal damping model it could however be different from $\Gamma_{\mathrm{PhC}}(\omega = \Omega_{\mathrm{PhC}})$ by a factor $\Omega_{\mathrm{PhC}}/\Omega_{\mathrm{M}}$. This factor can be regarded as a constant due to the small Γ_{M}. For the whole system, we can then write

$$\begin{pmatrix}\chi^{-1}_{\mathrm{PhC}}(\omega) - \alpha_c & \alpha_c\\ \alpha_c & \chi^{-1}_{\mathrm{M}} - \alpha_c\end{pmatrix}\begin{pmatrix}\delta z_{\mathrm{PhC}}(\omega)\\ \delta z_{\mathrm{M}}(\omega)\end{pmatrix} = \begin{pmatrix}F_{\mathrm{PhC}}(\omega)\\ F_{\mathrm{M}}(\omega)\end{pmatrix}. \tag{S4}$$

Inverting the matrix on the left-hand side we get

$$\chi = \begin{pmatrix} \left(\chi_{\mathrm{PhC}}^{-1} + \frac{\alpha_c}{\alpha_c \chi_{\mathrm{M}} - 1}\right)^{-1} & \left(\chi_{\mathrm{PhC}}^{-1} + \chi_{\mathrm{M}}^{-1} - \frac{\chi_{\mathrm{PhC}}^{-1}\chi_{\mathrm{M}}^{-1}}{\alpha}\right)^{-1} \\ \left(\chi_{\mathrm{PhC}}^{-1} + \chi_{\mathrm{M}}^{-1} - \frac{\chi_{\mathrm{PhC}}^{-1}\chi_{\mathrm{M}}^{-1}}{\alpha}\right)^{-1} & \left(\chi_{\mathrm{M}}^{-1} + \frac{\alpha_c}{\alpha_c \chi_{\mathrm{PhC}} - 1}\right)^{-1} \end{pmatrix}, \tag{S5}$$

$$\begin{pmatrix} \delta z_{\mathrm{PhC}}(\omega) \\ \delta z_{\mathrm{M}}(\omega) \end{pmatrix} = \chi(\omega) \begin{pmatrix} F_{\mathrm{PhC}}(\omega) \\ F_{\mathrm{M}}(\omega) \end{pmatrix}. \tag{S6}$$

While the two modes are coupled, for our device ($\Omega_{\mathrm{PhC}} \gg \Omega_{\mathrm{M}}$) we can still approximate them as separate modes around Ω_{M}

$$\delta z_{\mathrm{M}}(\omega) \approx \left(\chi_{\mathrm{M}}^{-1}(\omega) + \frac{\alpha_c}{\alpha_c \chi_{\mathrm{PhC}}(\omega) - 1}\right)^{-1} F_{\mathrm{M}}(\omega). \tag{S7}$$

as the off-diagonal terms are much smaller in magnitude. Then, for the mechanical resonator, the inverse of effective susceptibility becomes

$$1/\chi_{\mathrm{M}}^{\mathrm{eff}}(\omega) = \chi_{\mathrm{M}}^{-1}(\omega) + \frac{\alpha_c}{\alpha_c \chi_{\mathrm{PhC}}(\omega) - 1}. \tag{S8}$$

We now look at the imaginary part (the dissipation [8]) and evaluate around the frequency of the mechanical resonator

$$\mathrm{Im}\frac{1}{\chi_{\mathrm{M}}^{\mathrm{eff}}(\omega)} \approx -m_{\mathrm{M}}\left(\Gamma_{\mathrm{M}} + \frac{\alpha_c^2 \Gamma_{\mathrm{PhC}}/m_{\mathrm{M}} m_{\mathrm{PhC}}}{\left(\alpha_c/m_{\mathrm{PhC}} - (\Omega_{\mathrm{PhC}}^2 - \Omega_{\mathrm{M}}^2)\right)^2 + \Gamma_{\mathrm{PhC}}^2 \Omega_{\mathrm{M}}^2}\right)\omega. \tag{S9}$$

We can clearly see that the dissipation is increased by

$$\delta\Gamma_{\mathrm{M}} = \frac{\alpha_c^2 \Gamma_{\mathrm{PhC}}/m_{\mathrm{M}} m_{\mathrm{PhC}}}{\left(\alpha_c/m_{\mathrm{PhC}} - (\Omega_{\mathrm{PhC}}^2 - \Omega_{\mathrm{M}}^2)\right)^2 + \Gamma_{\mathrm{PhC}}^2 \Omega_{\mathrm{M}}^2} \approx \frac{\alpha_c^2}{m_1 m_2}\frac{\Gamma_{\mathrm{PhC}}}{\Omega_{\mathrm{PhC}}^4}. \tag{S10}$$

Our model shows that if there is any coupling between the mechanical resonator and the mechanics of the photonic crystal, the quality factor of the mechanical resonator is always reduced. The last approximation is appropriate for our devices since $\Omega_{\mathrm{PhC}} \approx 8\Omega_{\mathrm{M}}$ for the fundamental mechanical mode of the photonic crystal. Though the equation shows a $\Omega_{\mathrm{PhC}}^{-4}$ dependence, we would like to emphasis that Γ_{PhC} also depends on Ω_{PhC}, in particular for structural damping, ($\Gamma_{\mathrm{PhC}} \sim \Omega_{\mathrm{PhC}}^2$ [5–7]). Nevertheless, increasing the mechanical frequency of the photonic crystal reduces the dissipation degradation, and is one of the main reasons why the tethers are used. In order to verify this, we have also designed and assembled photonic crystals without the clamping tethers with $\Omega_{\mathrm{PhC}} \approx 3\Omega_{\mathrm{M}}$, and see a systematically larger degradation in the mechanical quality factor.

Optical bistability

Optical bistability can either be caused by photon absorption of the material that generates heat, or the static optomechanical effect [8, 9]. Since both similarly affect our measurements, we do not need to distinguish them. Furthermore, the extra clamping tethers we introduce can compensate for both as they increase the thermal conductivity and the rigidity of the photonic crystal structure.

In the presence of linear absorption or the static optomechanical effect, the optical resonance frequency shifts and is proportional to the cavity photon number with a coefficient β,

$$\delta\omega_{\mathrm{cav}} = \omega_{\mathrm{cav}} - \omega_{\mathrm{cav}}^{(0)} = \beta n_{\mathrm{cav}}(\Delta), \tag{S11}$$

where $\omega_{\mathrm{cav}}^{(0)}$ is the original cavity resonance frequency. In a typical situation, $\beta < 0$. The actual detuning, is then a function of the cavity photon number

$$\Delta(n_{\mathrm{cav}}) = \Delta_0 - \beta n_{\mathrm{cav}}(\Delta). \tag{S12}$$

Δ_0 is the detuning with vanishing input power and is a fixed value for a given laser wavelength. Let the cavity photon number at resonance be N_{cav}, which is given by

$$n_{\mathrm{cav}}(\Delta) = \frac{(\kappa/2)^2}{(\kappa/2)^2 + \Delta^2} N_{\mathrm{cav}}, \tag{S13}$$

for a fixed input power. Equation (S12) and (S13) define the behavior of the system, and we use them to fit the traces in Figure 4(a) in the main text. Combining these two equations, we get an equation for Δ,

$$\Delta^3 - \Delta_0 \Delta^2 + \left(\frac{\kappa}{2}\right)^2 \Delta + \left(\frac{\kappa}{2}\right)^2 (\beta N_{\mathrm{cav}} - \Delta_0) = 0 \tag{S14}$$

Optical bistability occurs when there are more than one possible $\Delta \in R$ for a fixed laser frequency, namely a fixed Δ_0. Consider a situation when the laser is on resonance, $\Delta = 0$. A small perturbation, either slightly reducing the photon number or Δ becoming slightly smaller than 0, the cavity photon number is reduced and hence the optical resonance frequency drifts to the high frequency side. This then reduces the cavity photon number and makes Δ more negative, forming a positive feedback loop. The system would then abruptly transit to a state with another Δ, if it exists, where the optical cavity resonance is very far away and the cavity photon number is small. The system would not come back to the original state with any further perturbations. To avoid this instability, it has been proposed to always work at an optical wavelength where multiple values for Δ are not possible [10]. However, as this is in the blue-detuned regime, it can lead to coherent driving and heating of the mechanical resonator, and the system becomes unstable again when a high Q_{M} mechanical resonator is involved. On the other

hand, if there is only one feasible Δ, the state of the system is still close to the original state. This process is continuous, and a small change of the environment can still bring the system back. Thus, the transition between multiple solutions and a single solution in equation (S14) marks the boundary between a stable and an unstable system.

Let us now define

$$y(\Delta) = \Delta^3 - \Delta_0\Delta^2 + \left(\frac{\kappa}{2}\right)^2 \Delta + \left(\frac{\kappa}{2}\right)^2 (\beta N_{\mathrm{cav}} - \Delta_0)\,. \tag{S15}$$

For equation (S14), having multiple solutions implies that y is not a monotonically increasing function and $y'(\Delta)$ should have 2 real solutions. Thus, $\Delta_0^2 - \frac{3}{4}\kappa^2 > 0$ and the local minimum and maximum happens when

$$\Delta = \Delta_\pm = \frac{1}{3}\Delta_0 \pm \frac{1}{6}\sqrt{4\Delta_0^2 - 3\kappa^2}. \tag{S16}$$

Then, the bistability occurs if and only if there exists a real ϵ_0 such that $y(\Delta_+) < 0$ and $y(\Delta_-) > 0$. Note that both $y(\Delta_+)$ and $y(\Delta_-)$ are monotonically decreasing as Δ_0 increases in their ranges. The bistability bound is then given by

$$N_{\mathrm{cav}} > \left|\frac{4\sqrt{3}\kappa}{9\beta}\right|. \tag{S17}$$

In the main text, the non-stable regime is colored in gray in Figure 4(b), with β obtained by the linear fit of the change of optical resonance frequency. We note that the measurement in the bistability regime might not be as reliable. We first perform a linear fit to all the data points in Figure 4(b), extracting a bistability bound of $N_{\mathrm{cav}} \approx 3000$. We then perform another fitting with only the data $N_{\mathrm{cav}} < 3000$ and do not see a substantial difference between the two fits. The fitting result and the corresponding bistability bound is shown in Figure 4(b).

Experimental method

Before assembling the structure, we measure the quality factor of the bare mechanical structure, by bringing a sharp single-ended conical optical fiber taper [4] above the center of the mechanical structure. When the distance is sufficiently close, the evanescent field couples to the mechanical motion. By first touching the mechanical structure with the fiber and then lifting it, the fundamental mode is excited, which allows us to perform a ringdown measurement.

Next, we move the devices to a different setup for assembly, which is described in the "assembling setup and procedure" section. As shown in Figure 2 in the main text, several parameters can in principle affect the final performance of the device after assembly. In this work, we focus on the effect of the gap size between the photonic crystal and the mechanical structure as it is possible to be controlled very precisely and consistently through the thickness of the spacers. In addition, the ability to achieve various gap sizes, in particular below 100 nm, marks the flexibility of our assembly technique.

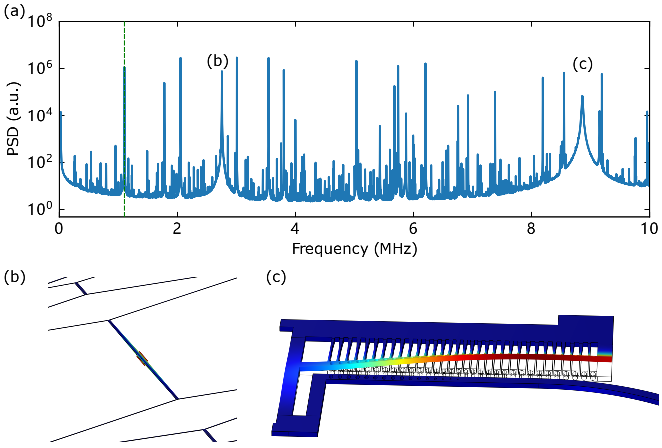

FIG. S3. (a) Mechanical spectrum over a large frequency range. The fundamental mode (marked in green) has a high mechanical quality factor and large optomechanical coupling to the optical cavity. (b) and (c) show a torsional mode of the mechanical structure and the fundamental mechanical mode of the photonic crystal, respectively.

After assembling the structure, we use the same fiber taper as used in the bare mechanical structure measurement to couple light into the waveguide next to the photonic crystal. The light then evanescently couples into the optical cavity [4]. The end of the waveguide is patterned with photonic crystals, which acts as a mirror and couples the light out from the cavity back into the fiber.

Exemplary data obtained from the device characterization is shown in Figure S4, including the spectrum close to the mechanical mode, a ringdown measurement for the mechanical resonator before (light color) and after (dark color) assembling the full device, an optical cavity reflection measurement and an optical spring measurement. From the fits we obtain a quality factor of $Q_{\mathrm{M}} = 1.49 \times 10^7$ and $g_0/2\pi = 257.4 \pm 4.9$ kHz.

Mechanical resonator design

The mechanical resonator is formed by a long string and a fractal structure, which is shown in Figure S5. On each level of the fractal, a set of parameters, including the tether widths, lengths, the size of the diamond-shape connections, are assigned. This forms a large parameter space and optimization is performed using the Python package Py-BOBYQA [11]. It implements a derivative-free optimization algorithm to minimize the objective function (mechanical quality factor) within a bounded

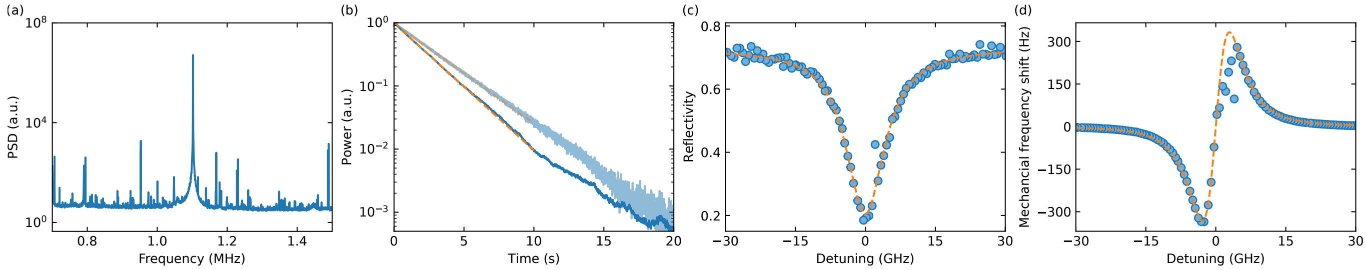

FIG. S4. Characterization of one of our devices. (a) Mechanical spectrum clearly showing the resonance peak of the fundamental mechanical mode at around $\Omega_{\rm M}/2\pi = 1.10$ MHz. (b) Ringdown measurements. The light color shows the result before assembling the device. The fit shows $Q_{\rm M} = 1.90\times10^7$. The dark color is the result of the assembled device, with $Q_{\rm M} = 1.49\times10^7$. (c) Normalize cavity reflection around the optical resonance (1554.6 nm). The fitting yields a linewidth $\kappa/2\pi = 10.1$ GHz. While the spectrum is normalized, it does include the inefficiency of the fiber-waveguide coupling. (d) Optical spring measurement by detuning the input laser around the cavity resonance frequency. On the blue detuning side ($\omega_{\rm L} > \omega_{\rm cav}$), the mechanical frequency is not stable due to optical heating, resulting in a slightly non-ideal fit to the data.

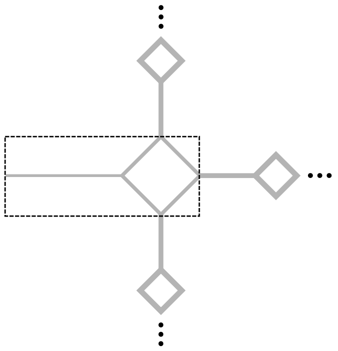

FIG. S5. Sketch of the fractal structure. The parent node with the main mechanical resonator (in the black box) is connected to 3 child nodes via the diamond shape formed by four tethers. Each child node is then connected to 3 tethers through another diamond shape, repeating the pattern 4 times for our particular design. The last level of diamonds are connected to the substrate via tethers at each of the vertices.

parameter space. It approximates a small region in the parameter space by a quadratic function, and then seeks the next point by interpolation. Due to the expensive simulation of the mechanics and the large parameter space, the optimization is terminated when the quality factor is "good enough". For the mechanical structure, it still has in-plane and torsional modes. Both can have low mechanical quality factor, and their thermal motion might present as excess classical noise in sensitive measurements. We thus also want the frequency of the fundamental in-plane mode and the torsional mode to be as far away in frequency as possible. This is however, not included in the optimization part and is performed manually by enlarging the center diamond.

$G_{\rm OM}$ calculation

The optomechanical coupling strength is given by $G_{\rm OM} = d\omega_{\rm cav}/dx$ [8], where $\omega_{\rm cav}$ is the resonance frequency of the optical cavity and x is the displacement of the mechanical resonator (defined as the displacement of the center of the mechanical beam in this work). For a small displacement, we use the method described in ref. [12] to evaluate the shift of the optical frequency, which then allows us to directly obtain $G_{\rm OM}$.

In Figure 2(d) of the main text, we see a linear dependence of $G_{\rm OM}$. As shown in ref. [12],

$$\frac{d\omega_{\rm cav}}{dx} \sim \int dA \left(\Delta\epsilon(|E_\parallel^{(\rm u)}|^2 - |E_\parallel^{(\rm l)}|^2) - \Delta(\epsilon^{-1})(|D_\perp^{\rm u}|^2 - |D_\perp^{\rm l}|^2)\right), \tag{S18}$$

where E is the electric field, $D = \epsilon E$ is the electric displacement field, ϵ is the relative permittivity, $\Delta\epsilon$ is the difference of the relative permittivity (between SiN and vacuum), and $\Delta(\epsilon^{-1})$ is the difference of the inverse of the relative permittivity. The integral is evaluated over the upper (u) and lower (l) surface of the mechanical structure. The symbols $\perp$ and $\parallel$ label the perpendicular and the parallel components of the field. Due to the small thickness (h) of the mechanical structure, by keeping the lowest order, $E_\parallel^{\rm u} \approx E_\parallel^{(\rm m)} + \frac{dE_\parallel}{dz}\frac{h}{2}$, $E_\parallel^{(\rm l)} \approx E_\parallel^{\rm m} - \frac{dE_\parallel}{dz}\frac{h}{2}$, where m stands for the middle plane of the mechanical structure, and z is for the z component in space. The same approximation applies to the D field. The difference is then approximated by the gradient of the electrical field. For example, the electric field part

$$(|E_\parallel^{(\rm u)}|^2 - |E_\parallel^{(\rm l)}|^2) \approx 2hE_\parallel^{(\rm m)}\frac{dE_\parallel}{dz}. \tag{S19}$$

Therefore, the optomechanical coupling is proportional to the thickness the mechanical structure, and it depends

on the gradient of the field across the structure. Intuitively, when the mechanical structure is moving, it should experience some changes of the electrical field in order to shift the optical resonance frequency. The constant part of the field would cancel out, since the upper and the lower surfaces are parallel and they move together. Then, to the lowest order and when the field is non-zero, for a thin beam, G_{OM} is approximately proportional to the integral of the thickness times the electrical field gradient.

Comparison to relevant work

**Ref.**	**Material**	**Motion direction**	$\kappa/2\pi$	$g_0/2\pi$	f_{M}	$\Gamma_{\mathrm{M}}/2\pi$	C_0	n_{cav}	n_{th}	C_{qu}
This work	SiN	Out of plane	10 GHz	257 kHz	1.1 MHz	0.074 Hz	3.6×10^2	3000	5.6×10^6	0.19
[13]	Si_3N_4	In plane	0.64 GHz	120 kHz	8 MHz					
[14]	Si_3N_4	In plane	77 GHz	358 kHz	0.94 MHz	1.76 Hz	3.8	734	6.5×10^6	4.3×10^{-4}
[2]	Si_3N_4	In plane	33 GHz	237 kHz	0.95 MHz	0.0035 Hz	196	120	6.5×10^6	3.6×10^{-3}
[15]	Si_3N_4	Out of plane	0.91 GHz	20 kHz	4.3 MHz	5.7 Hz	0.31	10^5	1.4×10^6	2.2×10^{-2}
[16]	Si	In plane	518 GHz	11.5 MHz	5 MHz	$< 10^4$	0.16			
[17]	Si	In plane	20.4 GHz	25 MHz	3.2 MHz	100 Hz	1.2×10^3			

TABLE I. Comparison of the parameters of our device to other relevant work. C_0: single photon cooperativity. n_{cav}: maximum cavity photon number in the experiment. n_{th}: thermal phonon number at room temperature (295 K). C_{qu}: quantum cooperativity at room temperature. In [15] and in [17], experiments were performed at cryogenic temperature (4.4 K and 3 K, respectively), and the room temperature data is not reported. To get extrapolate C_{qu} at room temperature, we assume that Γ_{M} is constant.

[1] R. A. Norte, M. Forsch, A. Wallucks, I. Marinković, and S. Gröblacher, Platform for measurements of the casimir force between two superconductors, Phys. Rev. Lett. **121**, 030405 (2018).
[2] J. Guo, R. Norte, and S. Gröblacher, Feedback Cooling of a Room Temperature Mechanical Oscillator close to its Motional Ground State, Phys. Rev. Lett. **123**, 223602 (2019).
[3] S. Gröblacher and R. Norte, High-selectivity dry release of dielectric structures (2021).
[4] M. J. Burek, C. Meuwly, R. E. Evans, M. K. Bhaskar, A. Sipahigil, S. Meesala, B. Machielse, D. D. Sukachev, C. T. Nguyen, J. L. Pacheco, E. Bielejec, M. D. Lukin, and M. Lončar, Fiber-coupled diamond quantum nanophotonic interface, Phys. Rev. Appl. **8**, 024026 (2017).
[5] P. R. Saulson, Thermal noise in mechanical experiments, Phys. Rev. D **42**, 2437 (1990).
[6] M. Imboden and P. Mohanty, Dissipation in nanoelectromechanical systems, Phys. Rep. **534**, 89 (2014).
[7] S. A. Fedorov, V. Sudhir, R. Schilling, H. Schütz, D. J. Wilson, and T. J. Kippenberg, Evidence for structural damping in a high-stress silicon nitride nanobeam and its implications for quantum optomechanics, Phys. Lett. A **382**, 2251 (2018).
[8] M. Aspelmeyer, T. J. Kippenberg, and F. Marquardt, Cavity optomechanics, Rev. Mod. Phys. **86**, 1391 (2014).
[9] V. R. Almeida and M. Lipson, Optical bistability on a silicon chip, Opt. Lett. **29**, 2387 (2004).
[10] J. Wang, K. P. Nayak, and J. Keloth, Photothermal tuning and stabilization of a photonic crystal nanofiber cavity, Opt. Lett. **44**, 3996 (2019).
[11] C. Cartis, J. Fiala, B. Marteau, and L. Roberts, Improving the flexibility and robustness of model-based derivative-free optimization solvers, ACM Trans. Math. Softw. **45**, 32 (2019).
[12] S. G. Johnson, M. Ibanescu, M. A. Skorobogatiy, O. Weisberg, J. D. Joannopoulos, and Y. Fink, Perturbation theory for Maxwell's equations with shifting material boundaries, Phys. Rev. E **65**, 066611 (2002).
[13] M. Eichenfield, R. Camacho, J. Chan, K. J. Vahala, and O. Painter, A picogram- and nanometre-scale photonic-crystal optomechanical cavity, Nature **459**, 550 (2009).
[14] A. G. Krause, T. D. Blasius, and O. Painter, Optical read out and feedback cooling of a nanostring optomechanical cavity, arXiv:1506.01249 (2015).
[15] D. J. Wilson, V. Sudhir, N. Piro, R. Schilling, A. Ghadimi, and T. J. Kippenberg, Measurement-based control of a mechanical oscillator at its thermal decoherence rate, Nature **524**, 325 (2015).
[16] R. Leijssen and E. Verhagen, Strong optomechanical interactions in a sliced photonic crystal nanobeam, Scientific Reports **5**, 15974 (2015).
[17] J. T. Muhonen, G. R. L. Gala, R. Leijssen, and E. Verhagen, State Preparation and Tomography of a Nanomechanical Resonator with Fast Light Pulses, Phys. Rev. Lett. **123**, 113601 (2019).
